# Book Notices

**Published:** 1884-02

**Authors:** 


### Book Notice.

There has just issued from the publishing house of P. Blakiston, Son & Co. "Dental Medicine. A Manual of Materia Medica and Therapeutics for Practitioners and Students," By Ferdinand J. S. Gorgas, A.M., M.D., D.D.S. This is the first work of any considerable extent upon this subject by an American author

Dr. J. W. White a few years ago issued a little work entitled "Dental Materia Medica." This, however, was little more than a presentation of the common therapeutic agents in general use by dentists, with some brief references to their properties and modes of employment. That was very good, for the time at least, but the profession has been growing, and something more extended is required now. The work on the same subject by Mr. James Stocken, L.D.S., of England, has been before the profession for several years, and has served a valuable purpose indeed. But it has been often suggested, recently at least, that a work of still wider range is demanded; and to the preparation of such a work Dr. Gorgas has devoted himself for the past year or two.

There is, perhaps, no one in this country better able to prepare such a manual than Prof. Gorgas. He has large experience in various directions, that has, in an eminent degree, qualified him for such a task. His course of instruction as a professor has, for twenty-five years, embraced the subject of Materia Medica, and in addition to this he has edited the later editions of Harris' Principles and Practice of Dental Surgery, and Harris' Dental Dictionary, and in addition to these he has been editor of the American Journal of Dental Science. The material in these ways gained, has been utilized in the production of this work, one which will be highly prized by the profession, and especially by dental students, who have hitherto had to rely mainly upon text books, prepared for medical students, upon this branch of study.

Space and time now forbid a review of the book; this we may make in a future number. The best method of reviewing it, however, is for every member of the dental profession to examine it for himself.

The work contains 311 pages. Price $3. It can be had of Robert Clarke & Co. and of booksellers generally.

